# Police Decision-Making in the Absence of Evidence-Based Guidelines: Assessment of Alcohol-Intoxicated Eyewitnesses

**DOI:** 10.3389/fpsyg.2022.761956

**Published:** 2022-02-03

**Authors:** Daniel Pettersson, Magnus Bergquist, Angelica V. Hagsand

**Affiliations:** Department of Psychology, University of Gothenburg, Gothenburg, Sweden

**Keywords:** police, decision-making, social norms, intoxication, witness, intervention

## Abstract

Regarding police procedures with alcohol-intoxicated witnesses, Swedish police officers have previously reported inconsistent and subjective decisions when interviewing these potentially vulnerable witnesses. Most officers have also highlighted the need for national policy guidelines aiding in conducting investigative interviews with intoxicated witnesses. The aims of the two studies presented here were to investigate whether (1) police officers’ inconsistent interview decisions are attributable to a lack of research-based knowledge; (2) their decision to interview, as well as their perceptions of the witnesses’ credibility could be influenced by scientific research; and (3) police officers decision-making and perceptions of witness credibility are biased by pre-existing social norms. In two separate randomized online experiments, police professionals and recruits (Study 1, *N* = 43; Study 2, *N* = 214) watched a recorded fictive witness interview to which they were asked to rate the probability of interviewing the witness, the witness’ credibility, and to estimate the witness’ level of intoxication. Results showed that interview probability and perceived witness credibility were affected by witness intoxication level. While it cannot be stated definitely from the present research, these findings provided indications that police officers and recruits lacked research-based knowledge. Results also showed that interview probability, but not perceptions of credibility, was influenced by a research-based message. In line with research, interview probability for the most intoxicated witness increased after reading the message. Unexpectedly, neither interview probability nor witness credibility was affected by social norms. The current findings added to the legal psychology literature by showing that a breath alcohol concentration (BrAC) as low as .04% was enough for police officers and recruits to consider intoxicated witnesses less credible than sober witnesses. Findings also indicated that, despite the lower credibility assessment, police may have some understanding that these witnesses can be interviewed at low intoxication levels (i.e., around .04%). However, this willingness to interview intoxicated witnesses ceased at a BrAC lower than the levels where research has found intoxicated witnesses as reliable as sober witnesses (i.e., BrAC < .10%). Future directions for research and policy development as well as theoretical and practical implications of the present findings are discussed.

## Introduction

In the past, the alcohol and memory literature have often found evidence of detrimental memory impairments being caused by alcohol-intoxication (e.g., Parker et al., 1976; Mintzer, 2007). This provides a rationale for the prevalent perception among legal practitioners (Kassin et al., 2001; Evans et al., 2009; Crossland et al., 2018; Hagsand et al., 2021, 2022; Monds et al., 2021a) and lay people (Evans and Schreiber Compo, 2010; Monds et al., 2021b) that intoxicated witnesses are less credible than sober witnesses. However, the effects of memory impairments caused by alcohol-intoxication have often not been replicated within the applied legal context of eyewitness memory (see Altman et al., 2019, for a review). When assessing intoxicated eyewitnesses’ memory, research has distinguished between the completeness of recall (i.e., total number of details recalled) and the accuracy of recall (i.e., number of correct details recalled; Schreiber Compo et al., 2019). A recent meta-analysis observed a dose-response relationship (i.e., memory impairments linearly increased with alcohol-intoxication) for completeness of recall but observed little effect of alcohol on the accuracy of intoxicated witnesses’ recall (see Jores et al., 2019), indicating the reliability of this witness group. Moreover, the effects of alcohol on eyewitness memory are nuanced and depending upon the intoxication level (see Altman et al., 2018 for an experimental bar study). Recent research show that intoxicated witness accounts often are reliable when breath alcohol concentration (BrAC) is approximately below .10%, with memory impairment increasing with higher levels of intoxication (see Altman et al., 2019, for a review). When interviews are conducted immediately after the witnessing of a crime, low to moderately intoxicated (BrAC < .10%) individuals reportedly are no less susceptible to suggestive leading questions than sober individuals (Mindthoff et al., 2021), and intoxicated persons also give complete and accurate statements (e.g., Hagsand et al., 2017; Mindthoff et al., 2019). In contrast, when the interview was postponed, both sober and intoxicated witnesses (e.g., Hagsand et al., 2017; Hildebrand Karlén et al., 2017; Schreiber Compo et al., 2017; Evans et al., 2018) gave less accurate information. Complete and accurate statements of eyewitnesses can be of central importance in criminal investigations (Kebbell and Milne, 1998), where criminal charges as well as subsequent court convictions often rely on evidence gathered from police interviews (Howe and Knott, 2015). Despite the known importance of obtaining information from witness interviews, police officers have reported inconsistent decisions to interview intoxicated witnesses and furthermore report varying procedures of engaging with them (i.e., waiting until witness is sober vs. interviewing immediately; Evans et al., 2009; Crossland et al., 2018; Monds et al., 2021a; Hagsand et al., 2022).

One way to further understand police officers’ inconsistent decisions to interview intoxicated witnesses has been studied through police surveys which were conducted both in Sweden and internationally (Evans et al., 2009; Crossland et al., 2018; Monds et al., 2021a; Hagsand et al., 2022). From these studies, several contributing factors to the officers’ inconsistent decisions can be derived. Firstly, many police officers perceived intoxicated witnesses as less credible than sober witnesses, which may have rendered them reluctant to interview these individuals (Evans et al., 2009; Crossland et al., 2018; Monds et al., 2021a; Hagsand et al., 2022). As the research described above has proposed otherwise, these findings suggested that police officers perhaps lacked research-based knowledge, highlighting the need for guidelines regarding intoxicated witnesses’ ability to accurately recall criminal events (Hagsand et al., 2022).

Secondly, procedural differences between officers as well as departments were evident across the studies mentioned above. The majority (69.9%) of UK police officers reported having different procedures for conduct with intoxicated and sober witnesses (Crossland et al., 2018), whereas approximately 40% of officers in Sweden (Hagsand et al., 2022) and 40% of officers in the US (Evans et al., 2009) reported different departmental procedures for these witness groups. In Sweden, the relatively low percentage of officers reporting different procedures could reflect the absence of an official research-based national policy that could provide guidelines with regards to proper conduct when encountering intoxicated witnesses.

A third contributing factor to the inconsistent interview decisions could be police officers self-reported reliance on observational methods to assess alcohol-intoxication (Evans et al., 2009; Crossland et al., 2018; Hagsand et al., 2022). Legality issues have commonly been cited as the reason for underutilized objective measurements (i.e., portable breathalyzer) when assessing witness intoxication levels. Instead of using objective measures of intoxication, officers reportedly make subjective assessments using observational methods (e.g., alcohol-odor, judging the behavior of the witness, using the standard field sobriety test, and conversational tests; Evans et al., 2009; Crossland et al., 2018; Hagsand et al., 2022). This raises the question of the accuracy of police officers’ judgments. A field study conducted in the United States reported a 98% accuracy in detecting alcohol-intoxication ≥.08% but showed a noticeable decrease to 71% accuracy for BrAC levels <.08% (Stuster, 2006). Another US study found that police officers, viewing a video clip, could not accurately decide if a person had even consumed alcohol until BrAC reached >.15% (Brick and Carpenter, 2001). The authors suggested that the visual stimulus used in their study left out important cues (e.g., alcohol-odor). Furthermore, a review on the use of observational methods for assessing alcohol-intoxication (e.g., alcohol-odor, standard field sobriety test, impaired walking, distorted speech, and finger to nose) concluded that all techniques were unsubstantiated (Rubenzer, 2011). These studies implied that while it is generally difficult to assess alcohol-intoxication through observation, it may be especially difficulty at low to moderate intoxication levels (i.e., BrAC <.10%; Stuster, 2006; Rubenzer, 2011).

Though not apparent in every study, these three contributing factors were all evident within the Swedish survey study by Hagsand et al. (2022), which clearly highlighted the necessity of disseminating research-based knowledge among Swedish law enforcement. Since 2019, police officers in the United Kingdom operate under research-based guidelines when they encounter intoxicated witnesses (College of Policing, 2019). Collaborations between researchers in Sweden and the Swedish Police Authority have begun, and the development of similar guidelines for intoxicated witnesses is underway (see Hagsand et al., 2020). However, this does not negate the fact that the police departments within Sweden and other countries currently still use inadequate methods of assessment when handling intoxicated witnesses. Indicative of this, 73% of police officers in Sweden (Hagsand et al., 2022), 74% of US officers (Evans et al., 2009), and 27% of officers in the United Kingdom (Crossland et al., 2018) reported that the decision to interview alcohol-intoxicated witnesses depended on the situation. For example, most Swedish police officers reported that they would consider the degree of the witness’ alcohol-intoxication before conducting an interview (Hagsand et al., 2022). Where the absence of guidelines, unapplied research-based knowledge and unsubstantiated methods to assess intoxication contribute to police’ behavior, a fourth factor may be the influence of social norms, generally applied in uncertain situations.

Social norms are informal rules shared by members of a particular social group and inform group members of behaviors and decisions (Cialdini and Trost, 1998; Legros and Cislaghi, 2020). These norms have been divided into descriptive and injunctive social norms, the former representing typical behaviors, the latter representing behaviors that are socially acceptable (Cialdini et al., 1990; Cialdini, 2012). A plethora of reasons exist for people’s compliance to social norms, some of which are out of a desire to hold accurate beliefs about the world, to maintain favorable concept of the self and with others (Cialdini and Goldstein, 2004), out of practicality (Anderson and Dunning, 2014), in anticipation of positive or negative social sanctions (Legros and Cislaghi, 2020), and importantly, as guidance in uncertain situations (Bell and Cox, 2015). The *focus theory* states that social norms must be focal in attention (i.e., made salient) if they are to effectively induce compliance (Cialdini et al., 1990). When combined, descriptive and injunctive norms can amplify the effect of normative influence on behavior (Miller and Prentice, 2016). However, when incongruous, the more focal norm would elicit compliance (Cialdini, 2012). Using social norms as a fourth contributing factor is of relevance to the current study, as these have shown to have a widespread and well-documented influence on behavior (e.g., Cialdini and Trost, 1998; Crano, 2000; Legros and Cislaghi, 2020) in contexts related to legal psychology, such as petty crimes (Keizer et al., 2008; Keuschnigg and Wolbring, 2015) and thievery (Cialdini et al., 2006). Descriptive social norms in particular have been found to influence police officer’s decision-making, where the knowledge of other officers intervening in a domestic violent situation through arrest increased the likelihood of police officers to do the same (Baldry and Pagliaro, 2014). Important to note is, however, that only those identifying strongly with their occupation were affected by the descriptive message.

This strong identification is often seen as a unique workplace culture, characterized by a strong sense of solidarity (Wieslander, 2019) and featured by external threats incongruent with most other workplaces (Marier and Moule, 2019). Because of this, group socialization processes, in which new recruits are encouraged to quickly adapt the tacit rules of the game (Gatto and Dambrun, 2012), have been argued to be especially strong within the Swedish Police Authority (Wieslander, 2019). These processes of socialization begin while recruits are still attending the Swedish Police Academy (Lander, 2013). As such, police culture develops professional and recruits alike. The information listed above would suggest that Swedish police officers, as well as the police officers within the other studies, could have been influenced by social norms for policing when assessing whether to interview intoxicated witnesses. This is an area within the field of legal psychology that has previously not been studied in light of police interviews with intoxicated witnesses and therefore represents a novel combination between the areas of legal psychology and social psychology.

The aims of the two studies presented here were to investigate whether (1) police officers’ inconsistent interview decisions are attributable to a lack of research-based knowledge; (2) their decision to interview, as well as their perceptions of the witnesses’ credibility could be influenced by scientific research; and (3) police officers decision-making and perceptions of witness credibility are biased by pre-existing social norms.

## Study 1

The purpose of Study 1 was to pilot-test the experimental procedure in a small sample of police officers and police recruits. An online experimental study using a mixed design was conducted. While there was no explicit test of participants research-based knowledge, the first aim was investigated by combining participants estimates of witness intoxication level, their stated interview probability, and their perception of witness credibility. The second aim was investigated by having participants read a short research-based message before responding to the dependent measures. The third aim was investigated using both a short descriptive normative message and by measuring levels of pre-existing descriptive and injunctive norms before they responded to the dependent measures.

### Materials and Methods

#### Participants

Professional police officers were recruited by invitations sent to all four regional police departments across Sweden. Police recruits were obtained by invitation sent to universities which managed police education in Sweden. A total of 84 participants clicked the invitation link. Some participants failed the attention checks (*n* = 11) or did not complete important study variables (*n* = 30) and were excluded. The final sample consisted of *N* = 43 participants. There was *n* = 17 (39.5%) police officers aged between 24 and 59 years old with almost an even split between men (52.9%) and women (47.1%). There was also *n* = 26 (60.5%) police recruits aged 19 to 45 years old, a majority of these were men (69%, women 31%).

#### Materials

The study was conducted online using the Qualtrics XM Platform. When starting the questionnaire, participants first viewed a short film (videos were inspired by Hirn Mueller et al., 2015, with the addition of having intoxicated witnesses) depicting a fictious eyewitness police interview with two actors (female witness and male interviewer) seated at a table in an interview room. In the scripted scenario, the woman had visited a bar with a friend, where she witnessed someone being stabbed in the stomach. The film depicted how the interviewer tried to elicit information from the witness regarding the event. There were three versions, each depicting the witness as either sober, moderately intoxicated, or highly intoxicated. The actor portraying the witness was given detailed instructions on how to simulate intoxication at the targeted BrAC levels (.00, .10, and .15%, respectively, for sober, moderate, and high intoxication levels), and the instructions were based on previous research concerning which behavior is present at different intoxication levels (Söderpalm, 2011). Examples of the instructions were as: the sober witness (0 alcoholic drinks) should not get distracted during the interview and should answer in a polite and straightforward way; the moderately intoxicated witness (approximately four alcoholic drinks) may be less focused, more easily distracted, with heightened emotions yet still in control; the highly intoxicated witness (approximately 8–12 alcoholic drinks) would be easily distracted, have somewhat slurred speech, be repetitive, act nauseous, and so forth. The only difference between the three films was the degree of witness alcohol-intoxication and each film was approximately 1 min in length.

#### Validation of the Stimulus Material

Estimates of witness intoxication were conducted first in a sample of psychology students from the University of Gothenburg, Sweden and additionally in a sample of Swedish police officers and recruits. The BrAC levels depicted in the films were .00% (sober), .10% (moderate), and .15% (high). Both university students and police officers were asked to estimate witness intoxication level on a 7-point Likert response format. Among both the university students (*N* = 102) and the police officers (*N* = 114), a significant effect of witness intoxication on estimates of intoxication was found, *F*_student_ (2,99) = 34.87, *p* < .001, $\eta_{p}^{2}$ = .41 and *F*_officer_ (2,111) = 35.73, *p* < .001, $\eta_{p}^{2}$ = .39. With university students, planned simple contrasts found that the highly intoxicated witness (*M* = 3.73, *SD* = 1.15) was estimated to be significantly more intoxicated compared to the moderately intoxicated (*M* = 2.51, *SD* = .79) and sober witnesses (*M* = 1.87, *SD* = .80, *p* < .001), and with a significant difference between the sober and the moderately intoxicated witnesses (*p* = .005). Similar effects were mirrored with police officers (*M*_high_ = 4.45, *SD*_high_ = 1.24; *M*_moderate_ = 2.81, *SD*_moderate_ = 1.18; *M*_sober_ = 2.17, *SD*_sober_ = 1.12), with a significant difference between the highly intoxicated witness and the sober and moderately intoxicated witnesses (*ps* < .001), and a significant difference between the sober and moderately intoxicated witnesses (*p* = .021). University students (*N* = 98) were also asked to estimate the BrAC level of the witness. A significant effect of witness intoxication on estimates of BrAC was found *F*(2,95) = 9.50, *p* < .001, $\eta_{p}^{2}$ = .17. Planned simple contrasts found that the highly intoxicated witness (*M* = .17, *SD* = .10) was estimated to be significantly more intoxicated compared with the moderately intoxicated (*M* = .10, *SD* = .07) and sober witnesses (*M* = .08, *SD* = .09, *p* < .001). Here, however, there was a non-significant difference between the sober and the moderately intoxicated witnesses (*p* = .566).

#### Procedure

Once participants gave their consent, they were given a battery of background questions. Thereafter, participants were randomized to one of four between-subject conditions: (1) control, (2) measured social norms, (3) induced descriptive social norm, and (4) research-based message. In the measured social norms condition, participants were asked about the extent to which they believed their colleagues would interview the witness and the extent to which their colleagues would approve if the participants themselves interviewed the witness. A 7-point Likert response format ranging from 1 = *none/disapprove* to 7 = *all/approve* was used to capture responses. In the induced descriptive norm condition, participants read that police officers considered intoxicated witnesses to be credible if BrAC is less than .10% and if open-ended questions were used.^1^ In the research-based message condition, participants read that research supported the view that intoxicated witnesses are credible if BrAC is less than .10% and if open-ended questions are used. All participants viewed all three films (depicting all intoxication levels) but in a counter-balanced order. All dependent variables were measured repeatedly after each film. Participants were asked (1) *How credible did you find the witness*? (2) *Would you interview the witness?* and (3) *How intoxicated do you think the witness were?* A 7-point Likert response format ranging from 1 = *Not at all/not likely at all/completely sober* to 7 = *Completely/most likely/extremely intoxicated* was used to capture responses. At the end of the study, attention checks were made.

#### Hypotheses

Based on previous research (Evans et al., 2009; Crossland et al., 2018; Monds et al., 2021a; Hagsand et al., 2022), we expected interview probability (H1a) and perceived witness credibility (H1b) to decrease as witness intoxication level increased. Due to potential lack of scientific knowledge among police officers, as suggested previously (e.g., Hagsand et al., 2022), we expected to find an effect of the research-based message on both interview probability (H2a) and perceived witness credibility (H2b). Based on the extensive research on social normative influence (e.g., Baldry and Pagliaro, 2014; Miller and Prentice, 2016), we expected to find an effect of the induced descriptive norm on both interview probability (H3a) and perceived witness credibility (H3b). We also expected to find an effect of activating pre-existing norms on both interview probability (H4a) and perceived witness credibility (H4b).

### Results

#### Manipulation Check of Witness Intoxication

There was a significant main effect of witness intoxication on police officers’ estimates of intoxication *F*(2,78) = 153.13, *p* < .001, $\eta_{p}^{2}$ = .80. Planned simple contrasts showed that participants considered the highly intoxicated witness to be significantly more intoxicated compared with the sober witness *F*(1,39) = 222.56, *p* < .001, $\eta_{p}^{2}$ = .85. There was no significant distinction between the sober and moderate witnesses *F*(1,39) = 1.54, *p* = .222, $\eta_{p}^{2}$ = .04. There was no main effect of information on intoxication estimate *F*(3,39) = .67, *p* = .576, $\eta_{p}^{2}$ = .05. Finally, there was a non-significant interaction effect of information and witness intoxication on intoxication estimate *F*(6,78) = .87, *p* = .520, $\eta_{p}^{2}$ = .06. Descriptive statistics are reported in Table 1. Results showed that participants estimated that the sober and moderately intoxicated witnesses were comparably intoxicated.

**Table 1:** Means (SD) for participants witness intoxication estimates in Study 1.

Witness intoxication	Information				Total *N* = 43
	Control *n* = 12	Social norm *n* = 12	Police norm *n* = 7	RBM *n* = 12	
Sober	2.08 (1.00)	2.33 (.89)	3.00 (1.00)	2.42 (1.51)	2.40 (1.14)
Moderate	2.42 (.90)	2.92 (.79)	2.86 (1.10)	2.50 (1.17)	2.65 (1.00)
High	5.17 (.72)	5.08 (.52)	5.29 (1.11)	4.83 (1.30)	5.07 (.91)

Participants were asked to estimate how intoxicated the witness were. Response format ranged from 1—completely sober to 7—extremely intoxicated. RBM, research-based message.

#### Main Analyses

For each dependent measure, a 4 (Information: control vs. measured norms vs. induced norm vs. research-based message) × 3 (Witness intoxication: sober vs. moderate vs. high) mixed design ANOVA’s with repeated measures on the second factor were conducted.^2^

#### Interview Probability

There was a significant main effect of witness intoxication on interview probability *F*(2,78) = 30.64, *p* < .001, $\eta_{p}^{2}$ = .44. Planned simple contrasts showed that participants were significantly less likely to interview the highly intoxicated witness compared with the sober witness *F*(1,39) = 30.36, *p* < .001, $\eta_{p}^{2}$ = .44; however, they made no significant distinction between the sober and moderate witnesses *F*(1,39) = 1.90, *p* = .176, $\eta_{p}^{2}$ = .05. This partially supported hypothesis 1a. There was a no main effect of information on interview probability *F*(3,39) = 1.23, *p* = .313, $\eta_{p}^{2}$ = .09. This contradicted hypotheses 2a, 3a, and 4a. Finally, there was no interaction between information and witness intoxication on interview probability *F*(6,78) = 2.15, *p* = .056, $\eta_{p}^{2}$ = .14. Descriptive statistics are reported in Table 2. Results showed that participants were just as likely to interview the sober witnesses as the moderately intoxicated witnesses.

**Table 2:** Means (SD) for interview probability in Study 1.

Witness intoxication	Information				Total *N* = 43
	Control *n* = 12	Social norm *n* = 12	Police norm *n* = 7	RBM *n* = 12	
Sober	6.42 (1.17)	6.83 (.58)	6.14 (1.07)	6.17 (1.27)	6.42 (1.10)
Moderate	6.75 (.62)	6.83 (.58)	6.43 (.98)	6.08 (1.34)	6.53 (.96)
High	4.50 (1.73)	6.08 (1.51)	4.86 (1.86)	5.25 (2.14)	5.21 (1.86)

Response format ranged from 1—not likely at all to 7—most likely. RBM, research-based message.

#### Witness Credibility

There was a significant main effect of witness intoxication on witness credibility *F*(2,78) = 35.97, *p* < .001, $\eta_{p}^{2}$ = .48. Planned simple contrasts showed that the highly intoxicated witness was rated significantly less credible than the sober witness *F*(1,39) = 37.84, *p* < .001, $\eta_{p}^{2}$ = .49 and the moderately intoxicated witness *F*(1,39) = 60.64, *p* < .001, $\eta_{p}^{2}$ = .61. However, there was no significant distinction between the sober and moderately intoxicated witnesses *F*(1,39) = .16, *p* = .691, $\eta_{p}^{2}$ = .00. This partially supported hypothesis 1b. There was no significant main effect of information on witness credibility *F*(3,39) = .47, *p* = .702, $\eta_{p}^{2}$ = .04. This contradicted hypotheses 2b, 3b, and 4b. Finally, there was a non-significant effect of witness intoxication interaction on witness credibility *F*(6,78) = .90, *p* = .502, $\eta_{p}^{2}$ = .06. Descriptive statistics are reported in Table 3. Results showed that participants perceived the sober and moderately intoxicated witnesses as comparably credible.

**Table 3:** Means (SD) for witness credibility in Study 1.

Witness intoxication	Information				Total *N* = 43
	Control *n* = 12	Social norm *n* = 12	Police norm *n* = 7	RBM *n* = 12	
Sober	5.25 (1.71)	5.25 (.62)	5.57 (1.13)	5.33 (1.37)	5.33 (1.25)
Moderate	5.25 (1.26)	5.00 (.60)	5.71 (.76)	5.67 (1.07)	5.37 (.98)
High	3.67 (1.37)	4.25 (.87)	4.43 (1.27)	4.25 (1.77)	4.12 (1.35)

Response format ranged from 1—not credible at all to 7—completely credible. RBM, research-based message.

### Discussion

The first aim of the present research was to investigate whether the previously reported (e.g., Evans et al., 2009; Hagsand et al., 2022) inconsistent interview decisions could be attributable to a lack of research-based knowledge. In the present context, we would conclude knowledgeability under three conditions: (1) if estimates of witness intoxication differed between the sober and moderately intoxicated witnesses, (2) if interview probability were comparable for the sober and moderately intoxicated witnesses but differed for the highly intoxicated witness, and (3) if credibility ratings were comparable for the sober and moderately intoxicated witnesses but differed for the highly intoxicated witness. Although no explicit test of research-based knowledge was used, if these three conditions were met it would be very likely that participants possessed knowledge of scientific research. The results showed comparable interview probability, and perceived witness credibility, for the sober and moderately intoxicated witnesses. The results also revealed a decrease in interview probability and perceived witness credibility for the highly intoxicated witness. In line with conditions 2 and 3, these results indicated that police officers’ and recruits’ decision and perception aligned with scientific research. However, contrasting condition 1, participants made similar intoxication estimates for the sober and moderately intoxicated witnesses. Therefore, the results remained inconclusive because there was no way to determine if participants treated the sober and moderate witnesses the same because they had pre-existing knowledge or because the degree of intoxication for these witnesses was considered similar.

The second aim of the present research was to investigate whether participants decision to interview, as well as their perceptions of the witnesses’ credibility, could be influenced by scientific research. Results showed that the research-based message had no significant impact on participants’ decision to interview the witnesses or on their perception of witness credibility. This indicated that a research-based message may not be a viable way to disseminate research findings among police officers. However, real-world policy guidelines like those used by police in the United Kingdom (College of Policing, 2019) are more extensive (i.e., a half page to one page) than a single sentence statement. It is possible that the short message used in Study 1 was insufficient to influence the participants. It is also possible that they already possessed this information and that the research-based message was not additionally helpful to them; however, results were inconclusive regarding participants knowledge base.

The third aim was to investigate whether police officer’s decision-making and perceptions of witness credibility were biased by social norms. Neither the induced descriptive norm nor the activation of pre-existing social norms influenced the interview decision or the perception of witness credibility.

## Study 2

In Study 1, it was not possible to determine if participants treated the sober and moderate witnesses the same because they had pre-existing knowledge or because they made similar intoxication estimates for these two witnesses. Thus, Study 1 remained inconclusive in terms of the first study aim. In Study 2, an attempt to untangle this issue was made by asking participants to estimate witness intoxication level (i.e., BrAC) rather than on a 7-point Likert response format. A related issue in Study 1 was that the approximately 1-min-long films may not have provided enough time for participants to observe the witness behaviors. Subtle but important mannerism changes between the sober and moderately intoxicated witnesses could have been difficult to detect. This may have contributed to the comparable intoxication estimates for the sober and moderate witnesses. Therefore, Study 2 included longer films to provide ample time to observe the witnesses.

Regarding the second study aim (i.e., whether participants decision to interview, as well as their perceptions of the witnesses’ credibility could be influenced by scientific research), Study 1 found no such prospect. It is possible that the short message in Study 1 was insufficient to influence participants. To strengthen the manipulation, a more extensive and real-world research-based message (see College of Policing, 2019, for actual UK guidelines) was used in Study 2.

In Study 1, social norms had no influence on participants’ decision and perception. One issue was that Study 1 did not account for how much participants identified with the reference group (i.e., other police and recruits). A strong identification with the reference group has been associated with a greater effect of a descriptive norm message (Baldry and Pagliaro, 2014; Liu et al., 2019). *Social identity theory* (Tajfel and Turner, 1979) states that in certain social contexts, people consider their group identity as more salient than their individual identity (Ellemers and Haslam, 2012). Consequently, people would be more likely to conform to social norms when there is a strong association between the individual and the group. Such a strong bond could be expected among police officers and recruits (Marier and Moule, 2019; Wieslander, 2019). Therefore, Study 2 included a measure of identification with the police occupation to investigate if the lack of social normative effect in Study 1 was related to social identity.

Overall, Study 1 was intended as a minor pilot study and had a smaller sample size which consequently meant lower power. Study 2 represented an improvement over Study 1 in several ways. In addition to collecting an adequate number of participants, the design in Study 2 was simplified. Since neither norm manipulation had any impact in Study 1 and the aim of the present research was initially to investigate pre-existing norms, the induced social norms condition (which was piloted in Study 1) was removed. This further simplified the research design. Moreover, the longer films meant participants could experience survey fatigue and therefore, a between-subjects design was used so each participant viewed only one film. The null findings in Study 1 prompted a revision of the response format used. Study 1 included a 7-point Likert format, but in order to increase sensitivity, Study 2 included a 10-point format.

### Materials and Methods

#### Participants

Police officers were recruited by invitation that was sent to all seven regional police departments across Sweden and *via* the national human resources department as well as personal contacts of the research team. In addition, all five universities which managed police education in Sweden was contacted *via* email and asked to forward an invitation to their police recruits. Finally, the invitation was also sent to police aspirants who underwent in-service training. A total of 336 people clicked the invitation link. Participants were excluded if they (a) did not consent (*n* = 8), (b) had participated in the pilot study (*n* = 2), (c) failed the attention check (*n* = 2), (d) did not complete the film viewing (*n* = 37), or (e) had missing data on all dependent measures and could not be analyzed (*n* = 73). Where data were available, attrition analyses showed that there was no significant gender difference between included and excluded participants (*p* = .473). Neither was there a significant difference in terms of how many police officers versus recruits were excluded (*p* = .908). Included and excluded police officers did not significantly differ in terms of experience working with witnesses (*p* = .126), and neither did recruits (*p* = .336). However, there was a significant mean difference in age [*t*(199.93) = −2.33, *p* = .02, Cohens *d* = .28]. Excluded participants (*M* = 35.59, *SD* = 9.77) were slightly younger than included participants (*M* = 38.56, *SD* = 11.20).

The final sample consisted of 214 participants. There was *n* = 152 (71%) professional police officers, a majority of these were men (men 57.2%, women 42.1%, and other .7%), and the average age was 42 years (*SD* = 11.12). Most (99.3%) professional police officers had experience interviewing witnesses (*M*_years_ = 13.16, *SD* = 10.89). All seven police regions in Sweden were represented in the sample (South 26.3%, West 20.4%, East 19.1%, Bergslagen 14.5%, Stockholm 12.5%, North 3.9%, Middle 3.3%). There was also *n* = 62 (29%) police recruits, a majority of these were men (men 79%, women 21%), and the average age was 32 years (*SD* = 7.82). Most recruits (74.1%) had been present for at least one witness interview. Most (80%) universities forwarded the invitation to their students (Linnaeus University; 30.6%, University of Borås; 29%, Södertörn University; 24.2%, and Malmö University; 16.1%).

#### Materials

To display more of the witness-interviewer interaction, the short films used in Study 1 were extended by editing together several films from the original set (inspired by Hirn Mueller et al., 2015) to create longer versions. The three edited films varied in length with the sober film playing 3 min, 43 s, the moderately, and highly intoxicated films, 3:51 and 4:26, respectively. Differences in seconds between the films were the cause of the instructions to the actor playing the witness (e.g., telling the actor to make slower responses and be more easily distracted). The films were validated and pre-study analyses are reported under materials for Study 1.

In summary, the research-based message^3^ stated that (1) level of intoxication greatly affects the extent of the memory impairments, (2) BrAC < .10% oftentimes does not affect witness memory, but in cases of negative effect, alcohol primarily affects the completeness of statements, and not the accuracy, (3) BrAC > .10% affects both completeness and accuracy, and (4) the most informative statements are obtained when witnesses are interviewed in close connection with the criminal event.

Inspired by previous research (Barreto and Ellemers, 2000; Baldry and Pagliaro, 2014), a short scale to measure social identity was constructed. It contained four propositional items (i.e., *being a member of the police is important to me, I feel like I am a part of the police, I feel good about being a part of the police, I feel the police occupation is the right fit for me*), presented in a 10-point Likert response format ranging from 1 = *completely disagree* to 10 = *completely agree*. A mean score across items was computed as a measure of identification with the police occupation (Cronbach’s *α* = .84).

#### Procedure

**Figure 1:**
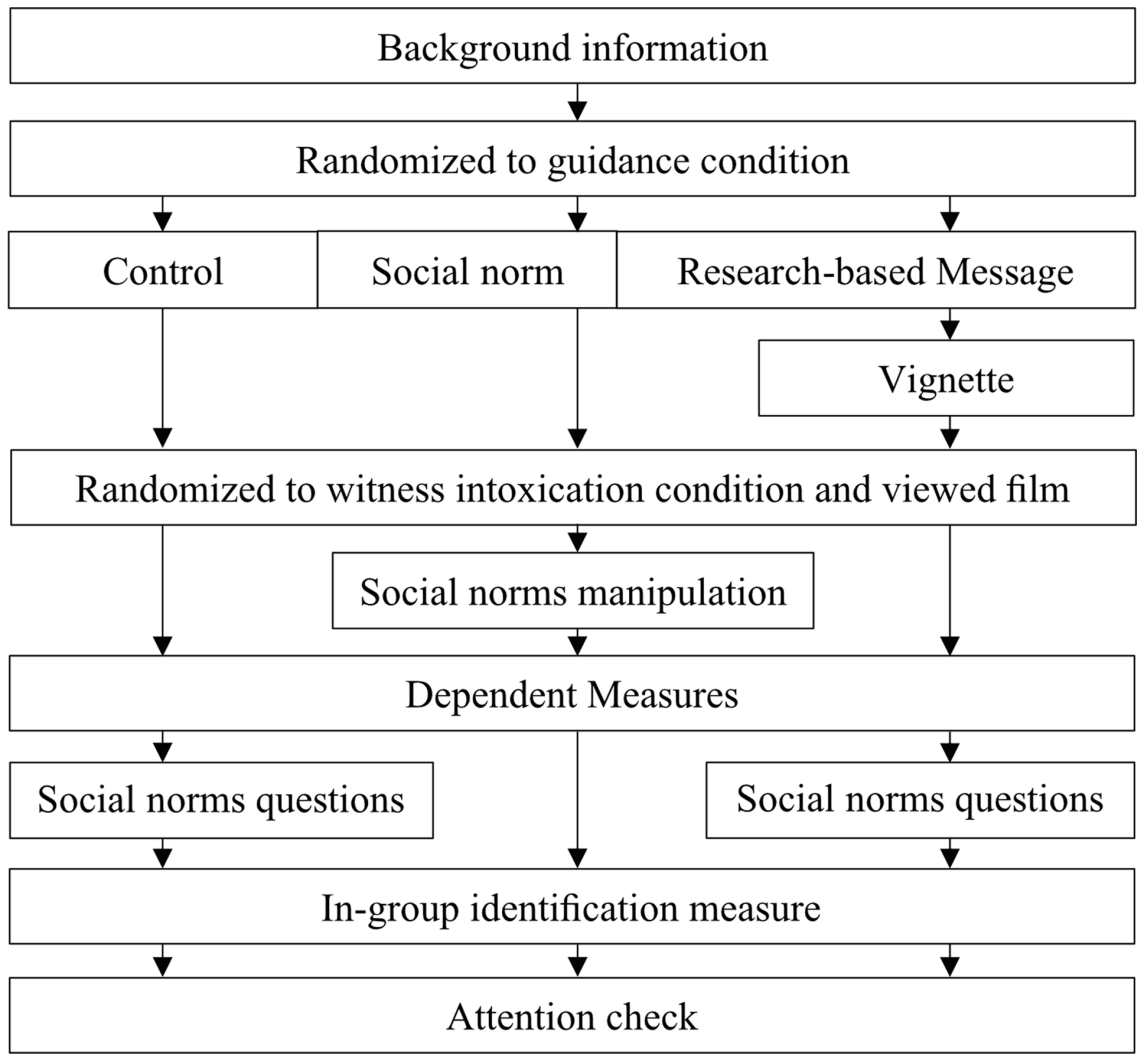
Overview of study procedure in Qualtrics.

Study 2^4^ was also conducted online using the Qualtrics XM Platform. Once they consented, participants were given a battery of background questions. They were then randomized to one of nine experimental conditions in a 3 (Information: control vs. social norm vs. research-based message) × 3 (Witness intoxication: sober vs. moderate vs. high) between-subjects experimental design (see Figure 1, for an overview of the study procedure in Qualtrics). Only participants in the research-based information condition read the research information after which participants in all conditions each saw one of the three films. After the film, only participants in the social norms condition were asked (in a 10-point Likert response format) the descriptive (i.e.*, on a scale from 1 to 10*, *how many of your police colleagues or fellow police students would interview the witness*?) and the injunctive (i.e., *on a scale from 1 to 10, would your police colleagues or fellow police students approve/disapprove if you interviewed the witness?*) norm activation questions. All participants were then asked the dependent measures of how credible they found the witness and how probable it was that they would conduct an interview. Responses were captured on 10-point response formats which ranged from 1 = *not credible at all/not likely at all* to 10 = *most credible/very likely*. Participants were also asked to estimate the witness BrAC on a two decimal continuum which ranged from 0 to 4 and presented to participants in per mile (‰).^5^ All participants were then asked how confident they were in their decision to interview the witness on a 10-point response format which ranged from 1—*not confident at all* to 10—*completely confident*. After this, participant in the control and research-based message conditions were asked the same social normative questions previously posed to participants in the social norms condition. All participants were then given the in-group identification measure. Finally, participants’ attention during the film viewing was checked by asking them to identify the event described by the witness from two possible scenarios (one sentence long each). The two options had a slight but salient difference so that participants who paid attention should be able to pick the right option without much difficulty.

#### Power Calculations

To form a basis for interpretation, the frequently referenced Cohen’s *d* guidelines which denoted a medium-sized effect as a mean difference of *d* = .50 (small *d* = .20, large *d* = .80; Cohen, 1988) was used. This was converted to Cohen’s *f* (small *f =* .10, medium *f* = .25, and large *f* = .40) for use with G^*^Power 3.1 (Faul et al., 2007) to calculate main and interaction effects for ANOVA. If there were any significant effects to be found, Study 2 (*N* = 214, *α* = .05) had a power of 91% to observe significant medium-sized main effects and a power of 84% to observe significant medium-sized two-way interaction effects.

#### Hypotheses

Based on the content of the research-based message (i.e., witnesses are generally reliable when BrAC is less than .10%) and the potential lack of research-based knowledge, we expected an interaction between information and witness intoxication. Compared to participants who received no information (i.e., control), participants who read the research-based message would be more likely to interview the moderately intoxicated witness compared to the sober witness (H1a) and less likely to interview the highly intoxicated witness compared to the sober witness (H1b). A similar interaction was expected for perceived witness credibility. We hypothesized that participants who read the research-based message (compared to participants in the control condition who received no information) would perceive the moderately intoxicated witness as more credible compared with the sober witness (H2a) and the highly intoxicated witness as less credible compared to the sober witness (H2b).

Based on the previous research demonstrating the effects of social normative influence (e.g., Miller and Prentice, 2016) and research which has found that police officers believed intoxicated witnesses were less credible than sober witnesses (Evans et al., 2009; Crossland et al., 2018; Monds et al., 2021a; Hagsand et al., 2022) as well as the potential lack of knowledge suggested previously (e.g., Hagsand et al., 2022), we predicted the following hypotheses for social norms. Compared to participants who received no information (i.e., control), participants for whom pre-existing social norms were activated (i.e., considering what their colleagues would do or approve of doing) would be less likely to interview the moderately intoxicated (H3a) and the highly intoxicated (H3b) witness compared to the sober witness. We expected similar main effects for perceived witness credibility. Compared to those who received no information (i.e., control), participants for whom pre-existing social norms were activated would perceive the moderately intoxicated witness (H4a) and the highly intoxicated witness (H4b) as less credible compared to the sober witness.

### Results

#### Manipulation Check of Social Norms

A strong positive correlation (*r* = .68, *p* < .001) between the descriptive and injunctive social norms measures indicated that what participants believed others would do and approve of doing, aligned well. A robust one-way ANOVA was conducted with information condition as independent variable and descriptive norm as dependent variable. There was a non-significant effect of information on descriptive norm [*F*(2,204) = 1.53, *p* = .22, *η*^2^ = .02], indicating a shared view among participants regarding the actions of others. Another robust one-way ANOVA found a significant effect of information on the injunctive norm [*F*(2,203) = 3.28, *p* = .04, *η*^2^ = .03]. *Post-hoc* comparisons showed a non-significant difference between the control (*M* = 7.67, *SD* = 2.65) and social norms conditions (*M* = 7.37, *SD* = 3.46, *p* = .85, *g* = .10, 95% CI [−.24, .44]). There was also a non-significant difference between the control and research-based message conditions (*M* = 8.53, *SD* = 2.10, *p* = .09, *g* = .36, 95% CI [.03, .70]). However, there was a significant difference between the social norms and research-based message conditions (*p* = .05, *g* = .41, 95% CI [.07, .75]). Results indicated that participants in the research-based message condition, to a larger degree, believed that others would approve of them interviewing the witness. This was unsurprising given that the research-based message contained information on the reliability of intoxicated witnesses.

#### Manipulation Check of Witness Intoxication

There was a significant main effect of witness intoxication on BrAC estimates [*F*(2,201) = 87.64, *p* < .001, $\eta_{p}^{2}$ = .47]. Bonferroni adjusted planned comparison showed a significant difference between the sober and the moderate conditions (*p* < .001, *g* = .67, 95% CI [.33, 1.01]), between the sober and high conditions (*p* < .001, *g* = 1.97, 95% CI [1.56, 2.38]), and between the moderate and high conditions (*p* < .001, *g* = 1.42, 95% CI [1.06, 1.79]). There was a non-significant effect of information on BrAC estimates [*F*(2,201) = 2.40, *p* = .094, $\eta_{p}^{2}$ = .02]. There was also a non-significant interaction effect of information and witness intoxication on BrAC estimate [*F*(4,201) = .18, *p* = .950, $\eta_{p}^{2}$ = .00]. Descriptive statistics are reported in Table 4. The results showed that participants clearly distinguished between all witness intoxication levels independent of information condition. An issue that is important to immediately notice is that participants estimated that the highly intoxicated witness had an average BrAC of .09% (see Table 4). This is much lower than the expected.15% and has consequences for the interpretation of the main analyses.

**Table 4:** Means (SD) for participants BrAC (%) estimates in Study 2.

Witness intoxication	Information			Total
	Control	Social norm	RBM	
Sober	*n* = 18 .01 (.02)	*n* = 24 .02 (.04)	*n* = 23 .02 (.02)	*n* = 65 .02 (.03)
Moderate	*n* = 30 .04 (.03)	*n* = 20 .05 (.03)	*n* = 25 .05 (.03)	*n* = 75 .04 (.03)
High	*n* = 24 .08 (.04)	*n* = 20 .10 (.05)	*n* = 26 .10 (.03)	*n* = 70 .09 (.04)
Total	*n* = 72 .05 (.04)	*n* = 64 .05 (.05)	*n* = 74 .06 (.04)	*N* = 210 .05 (.04)

Responses were captured on a continuous scale ranging from 0 to 4. RBM, research-based message.

#### Main Analyses

For each dependent measures, a 3 (Information: control vs. social norms vs. research-based message) × 3 (Witness intoxication: sober vs. moderate vs. highly intoxicated) between-subjects factorial ANOVA was conducted^6^ ^,^^7^.

#### Interview Probability

Descriptive statistics are reported in Table 5. There was a significant main effect of witness intoxication on interview probability [*F*(2,205) = 9.67, *p* < .001, $\eta_{p}^{2}$ = .09]. Bonferroni adjusted planned comparisons showed a non-significant difference between the sober and moderate conditions (*p* = .07, *g* = .43, 95% CI [.09, .76]), a significant difference between the sober and high conditions (*p* < .001, *g* = .72, 95% CI [.37, 1.06]), and a non-significant difference between the moderate and high conditions (*p* = .08, *g* = .33, 95% CI [.01, .66]). Participants were least likely to interview the highly intoxicated witness and most likely to interview the sober witness. There was also a significant main effect of information on interview probability, [*F*(2,205) = 3.80, *p* = .02, $\eta_{p}^{2}$ = .04]. Planned contrasts showed a non-significant difference between the control and social norm conditions (*p* = .33, *g* = .20, 95% CI [−.13, .54]); therefore, hypotheses 3a and 3b were not supported. There was a significant difference between the control and research-based message conditions (*p* = .007, *g* = .49, 95% CI [.17, .82]). Results showed that a research-based message, but not social norms, affected the interview probability. There was a non-significant information by witness intoxication interaction on interview probability [*F*(4,205) = 1.24, *p* = .29, $\eta_{p}^{2}$ = .02]; therefore, hypotheses 1a and 1b were not supported. Despite a non-significant interaction, visual inspection of the data showed a convergence of the slopes which indicated that in the research-based message condition only, witness intoxication had no effect. Unplanned simple effects analysis confirmed this research-based message [*F*(2,205) = .44, *p* = .64], control [*F*(2,205) = 5.40, *p* < .01], and social norm [*F*(2,205) = 6.06, *p* < .01].

**Table 5:** Means (SD) for interview probability in Study 2.

Witness intoxication	Information			Total
	Control	Social norm	RBM	
Sober	*n* = 18 8.22 (3.15)	*n* = 24 9.00 (2.57)	*n* = 23 8.48 (2.31)	*n* = 65 8.60 (2.64)
Moderate	*n* = 30 6.90 (3.32)	*n* = 22 7.00 (3.31)	*n* = 25 8.32 (1.87)	*n* = 77 7.39 (2.96)
High	*n* = 25 5.12 (3.60)	*n* = 21 5.81 (4.09)	*n* = 26 7.69 (3.33)	*n* = 72 6.25 (3.78)
Total	*n* = 73 6.62 (3.54)	*n* = 67 7.34 (3.56)	*n* = 74 8.15 (2.58)	*N* = 214 7.37 (3.30)

Response format ranged from 1—not likely at all to 10—very likely. RBM, research-based message.

#### Witness Credibility

Descriptive statistics are reported in Table 6. There was a significant main effect of witness intoxication on witness credibility [*F*(2,205) = 17.06, *p* < .001, $\eta_{p}^{2}$ = .14]. Bonferroni adjusted planned comparisons showed a significant difference between the sober and moderate conditions (*p* = .02, *g* = .52, 95% CI [.18, .86]), a significant difference between the sober and high conditions (*p* < .001, *g* = .95, 95% CI [.60, 1.30]), and a significant difference between the moderate and high conditions (*p* = .01, *g* = .45, 95% CI [.12, .77]). Results showed that participants rated the sober witness the most credible and the highly intoxicated witness the least credible. There was a non-significant main effect of information on witness credibility, [*F*(2,205) = 2.53, *p* = .08, $\eta_{p}^{2}$ = .02]. Planned contrasts showed a non-significant difference between the control and social norm conditions (*p* = .186, *g* = .13, 95% CI [−.20, .46]); therefore, hypotheses 4a and 4b were not supported. There was a non-significant difference between the control and research-based message conditions (*p* = .364, *g* = .18, 95% CI [−.15, .50]). Results showed that neither research-based message, nor social norms, affected witness credibility ratings. There was a non-significant interaction effect of information and witness intoxication on witness credibility [*F*(4,205) = 1.96, *p* = .102, $\eta_{p}^{2}$ = .04], and therefore, hypotheses 2a and 2b were not supported.

**Table 6:** Means (SD) for witness credibility in Study 2.

Witness intoxication	Information			Total
	Control	Social norm	RBM	
Sober	*n* = 18 7.94 (1.31)	*n* = 24 8.04 (1.46)	*n* = 23 7.78 (1.45)	*n* = 65 7.92 (1.40)
Moderate	*n* = 30 7.23 (2.00)	*n* = 22 6.68 (1.49)	*n* = 25 7.24 (1.74)	*n* = 77 7.08 (1.78)
High	*n* = 25 6.08 (2.02)	*n* = 21 5.33 (2.18)	*n* = 26 7.04 (1.87)	*n* = 72 6.21 (2.10)
Total	*n* = 73 7.01 (1.98)	*n* = 67 6.75 (2.03)	*n* = 74 7.34 (1.71)	*N* = 214 7.04 (1.91)

Response format ranged from 1— not credible at all to 10—completely credible. RBM = research-based message.

### Supplementary Analyses

#### Social Identity

Inspired by previous research (Baldry and Pagliaro, 2014), a median split (*Md* = 9.00) divided participants into *low identifiers* (*M* = 7.48, *SD* = 3.13) and *high identifiers* (*M* = 7.70, *SD* = 3.15) with respect to social identity. A 3 (Information: control vs. social norm vs. research-based message) × 2 (Social identification: low vs. high) between-subjects factorial ANOVA with the probability of interviewing the witness as dependent variable was conducted. There was a non-significant main effect of information on interview probability [*F*(2,181) = 2.01, *p* = .14, $\eta_{p}^{2}$ = .02]. There was a non-significant main effect of identification on interview probability [*F*(1,181) = .16, *p* = .69, $\eta_{p}^{2}$ = .00]. Finally, there was also a non-significant interaction effect of information and social identification on interview probability [*F*(2,181) = 1.32, *p* = .27, $\eta_{p}^{2}$ = .01]. Descriptive statistics are reported in Table 7. Results showed that identification with the police occupation had no impact on the probability of interviewing a witness, nor were participants who identified strongly with the police occupation influenced by social norms to a greater degree than those who identified less strongly.

**Table 7:** Means (SD) for identification with the police occupation in Study 2.

Information	Social identity	
	Low identifiers	High identifiers
Control	*n* = 27 6.63 (3.44)	*n* = 31 7.71 (2.91)
Social norm	*n* = 35 7.66 (3.36)	*n* = 28 6.89 (3.76)
Research-based message	*n* = 29 8.07 (2.37)	*n* = 37 8.30 (2.76)
Total	*n* = 91 7.48 (3.13)	*n* = 96 7.70 (3.16)

Composite score of the four-item scale. Response format ranged from 1—completely disagree to 10—completely agree.

#### Confidence Rating

A robust one-way ANOVA was conducted with witness intoxication level as independent variable and participants’ confidence in their decision to interview the witness as the dependent variable. There was non-significant effect of witness intoxication on participants confidence ratings {*F*(2,207) = 1.11, *p* = .33, *η^2^* = .01, 95% CI [.00, .05]}. Descriptive statistics are reported in Table 8. Results showed that participants who saw the film with the highly intoxicated witness were no less confident in their decision to interview than those who saw the sober and moderate witnesses.

**Table 8:** Means and standard deviations for confidence ratings across witness intoxication in Study 2.

Witness intoxication	*n*	*M*	*SD*
Sober	63	8.79	1.89
Moderate	76	8.25	2.33
High	71	8.38	2.41
Total	210	8.46	2.24

Table shows descriptive statistics for participants’ confidence in their decision to interview the witness across witness intoxication level. Response format ranged from 1—not confident at all to 10—absolutely confident.

### Discussion

The first aim of Study 2 was the same as in Study 1 to investigate whether police officers’ inconsistent interview decisions could be attributed to a lack of research-based knowledge. Again, there was no explicit test of police officers and recruit’s knowledge, instead such a conclusion, would be based on participants’ behavior when responding to the questions. In Study 2, there was the additional concern that police officers and recruits estimated that the highly intoxicated witness had an average BrAC of .09% (see Table 4), which was much lower than the intended .15%. This means that participants based their answers to the questions on a BrAC level in the low to moderate range (i.e., BrAC < .10%). Therefore, any further interpretation of the results must account for this lower estimate. Because of this, in Study 2, a lack of knowledge would be concluded if (1) interview probability differed across witness intoxication level, even for the “highly” intoxicated witness, and (2) if perceived witness credibility differed across witness intoxication level, again even for the “highly” intoxicated witness. Contrary to Study 1, participants made clear distinctions between all three witnesses’ intoxication levels. Interview probability remained the same for the sober and moderately intoxicated witnesses but differed significantly for the “highly” intoxicated witness. Perceived witness credibility significantly differed across all three levels of intoxication. Had participants possessed research-based knowledge, it should have been unlikely that they would have treated any of the witnesses differently because they all were estimated by the participants to have a BrAC level in the low to moderate range. A range where scientific research has found that intoxicated witnesses can be reliable (see Altman et al., 2019; Jores et al., 2019, for reviews and meta-analysis) and where the consequences of postponing an interview could lead to less complete and accurate statements (e.g., Hagsand et al., 2017; Hildebrand Karlén et al., 2017).

The second aim of Study 2 was again the same as in Study 1, to investigate whether their decision to interview, as well as their perceptions of the witnesses’ credibility could be influenced by scientific research. In line with Study 1, perception of witness credibility was unaffected by the research-based message. In contrast with Study 1, Study 2 found that the highly intoxicated witness was more likely to be interviewed compared with the condition that received no information (i.e., control). The research-based message informed participants about research regarding the reliability of low to moderately intoxicated witness statements and the consequences of postponing the interview. As such, it was unexpected to find an increase in interview probability for the highly intoxicated witness. However, when accounting for participants inaccurate estimates of intoxication level, these results made sense. The highly intoxicated witness was considered by participants to be in the low to moderate range and therefore encompassed by the information in the message. These findings, therefore, indicated that a research-based message might assist police officers and recruits to make decisions that are more in line with research findings. Such a message is more likely to affect the decision to interview than it is to affect perceptions of witness credibility.

The third aim was again to investigate whether police officers’ decision-making and perceptions of witness credibility are biased by pre-existing social norms. In line with Study 1, there was no statistically significant effect of social norms on interview probability in Study 2. People tend to comply with social norms more in uncertain situations where the right course of action is unclear (Bell and Cox, 2015). However, confidence ratings showed that all participants, regardless of witness intoxication level, were comparably confident in their decision to interview. Without the element of uncertainty, there may have been little reason for participants to look to others for guidance which may have diminished the impact of social norms. On the other hand, identification with the police occupation was high across all conditions which should have made compliance with the norm more likely (Baldry and Pagliaro, 2014; Liu et al., 2019).

## General Discussion

In recent surveys, Swedish police officers reported inconsistent individual interview decisions, absent policy guidelines, and subjective methods for assessing intoxication level among witnesses, victims, and suspects (Hagsand et al., 2021, 2022). Officers also reported perceptions of credibility contrary to research on this witness group (see Altman et al., 2019; Jores et al., 2019, for reviews and meta-analysis). This may produce uncertain situations in which the decision to interview might be unjustly influenced by social norms. Therefore, two studies were conducted to investigate whether (1) police officers’ inconsistent interview decisions are attributable to a lack of research-based knowledge; (2) their decision to interview, as well as their perceptions of the witnesses’ credibility could be influenced by scientific research; and (3) police officers decision-making and perceptions of witness credibility are biased by pre-existing social norms.

Prior to discussing the findings, it is necessary to mention again that participants’ estimation of intoxication level did not align with the pre-study validation of the stimulus material. In Study 2, participants inaccurately perceived both intoxicated witnesses to be low to moderately intoxicated. It is interesting to note that the university students in Study 1 made a far more accurate assessments about the highly intoxicated witness than the police officers in Study 2. Students were, however, far less accurate when assessing the sober witness compared to police officers in Study 2. Explanations addressing these issues surround the discussion of whether video clips are sufficient for making accurate estimates (Brick and Carpenter, 2001), and whether inaccuracies stem from using observational methods for assessing intoxication level which are ineffective (Rubenzer, 2011). Both the moderately and the highly intoxicated witnesses were estimated lower than what was intended in the research design, which could suggest the police frequent encounters with intoxicated people—many who are heavy drinkers (Evans et al., 2009; Crossland et al., 2018; Monds et al., 2021a; Hagsand et al., 2022), may have desensitized them to the behavioral effects of alcohol-intoxication. This might have resulted in the fact that no witness was perceived to be highly intoxicated by the police officers and recruits in Study 2. Any interpretations and implications that are made from the results therefore, must treat witnesses only from a sober to moderate intoxication level (i.e., <.10%), as these were the levels upon which participants based their answers to the survey questions.

The first aim was to investigate whether the previously reported inconsistent interview decision could be attributed to a lack of research-based knowledge (see also Hagsand et al., 2021, 2022). Although we did not explicitly test participants knowledge regarding research findings, we find it reasonable to expect cognizant police officers and recruits to consider low to moderately intoxicated witnesses comparably credible to sober witness, not hesitating to interview the former as much as the latter. This would be in line with research findings (e.g., Altman et al., 2019; Jores et al., 2019). Because all witnesses in Study 2 were considered low to moderately intoxicated, there should have been no variation in interview probability or perceived credibility across intoxication levels. However, participants rated the previously deemed highly intoxicated witness as less likely to be interviewed and less credible compared with the others. In addition, an unplanned simple effects analysis showed that after reading the research-based message about the reliability of low to moderately intoxicated witnesses, interview probability was less affected by degree of intoxication. This difference notes that participants did not make judgments based on pervious knowledge. These findings support previously self-reported survey results (e.g., Hagsand et al., 2022) as well as research which has found that police officers regarded intoxicated witnesses as less credible compared with sober witnesses (Evans et al., 2009; Crossland et al., 2018; Monds et al., 2021a; Hagsand et al., 2022). From the present data, it cannot be concluded whether police officers and recruits lacked prior knowledge in making judgments, but our results would favor the inference that this was in fact the case.

The second aim was to investigate whether their decision to interview, as well as their perceptions of the witnesses’ credibility could be influenced by scientific research. What both studies found was that perceived witness credibility remained unaffected by the research-based message. That is, regardless of the research-based information participants received, they were not influenced in their credibility judgments of the witnesses. However, in Study 2, the research-based message did influence participant’s willingness to interview the highly intoxicated witness, which was not the case in Study 1. The more informative message used in Study 2 could account for this discrepancy between studies, as a one sentence long message was perhaps not sufficient to affect participants in Study 1. Unexpectedly, the moderately intoxicated witness, who was estimated to have an average BrAC of .04%, was not more likely to be interviewed compared to the sober witness. A possibility is that police officers and recruits did not consider such a low intoxication level a reason to postpone the interview. As such, the research-based message was not additionally helpful to them. These findings indicated that police officers and recruits may have a basic understanding that witnesses can be interviewed at low levels of alcohol-intoxication (i.e., around .04%), but that they believe that this ceases to be the case at a lower intoxication level than what scientific research has suggested. It is interesting to note that the decision to interview increased after reading the message, even though participants still considered both intoxicated witnesses to be less credible than the sober witness. It appears that the decision to interview was made despite internally held perceptions. Perhaps participants were more affected by the information regarding the consequences of postponing the interview than they were by the information about intoxicated witness reliability. As such, they may have decided to interview the witness to avoid losing important details to a crime but remained confident that intoxicated witnesses are less credible. In summary, a research-based message may be a key method to encourage the right procedure when deciding to interview an intoxicated witness. In addition, and in concurrence with previous literature, the results showed the tendency of the police to perceive witnesses as less credible, even with BrAC as low as .04% (as the current study has found; Evans et al., 2009; Crossland et al., 2018; Monds et al., 2021a; Hagsand et al., 2022).

The third aim was to investigate whether police officers’ decision-making and perceptions of witness credibility are biased by pre-existing social norms. Neither study found that participants were biased by social normative influence, neither in their interview decision nor their estimates of witness credibility. Participants who were prompted to think about injunctive and descriptive norms were comparable to those who were not stimulated by such norms, and this trend was consistent across all intoxication conditions. The results seem to infer that social norm had little impact on both the decision to interview a witness, and perceived credibility. Considering the abundance of the general literature demonstrating social normative influence in various behaviors and contexts, these findings were unexpected (e.g., Rivis and Sheeran, 2003; Melnyk et al., 2010; Fischer et al., 2011; Baldry and Pagliaro, 2014; Bergquist et al., 2019), but more research within the police context is needed.

As a possible explanation for the null findings of social norms in Study 1, Study 2 included a measure of identification with the police occupation. *Social identity theory* (Tajfel and Turner, 1979) has suggested that a strong sense of in-group identification will incite people to act more in line with their group identity than their individual identity, resulting in social norm influences being particularly effective when the group identification is strong. In contrast with previous research (Baldry and Pagliaro, 2014; Liu et al., 2019), the degree of identification with the police occupation did not impact the effect of social norms in Study 2. In addition, confidence in their decision to interview the witness remained the same across intoxication levels. All participants strongly identified with the police occupation which (apart from indicating possible ceiling effects) should have increased the social normative influence. Having the questionnaire at the end could have impacted the study in two competing ways. First, the study procedure itself could have made their police identities salient before they answered the questionnaire, which would explain the high average. Second, social norms remained non-significant, which perhaps indicated that their identities were not salient when they answered the dependent measures. Had the identification questions been included earlier in the study the participants’ identities could have been salient when they made their decisions regarding the witnesses and the study procedure could not have affected their identification responses. As previously stated, social norms must be activated to influence decisions and behaviors (Cialdini et al., 1990), and they have a greater influence on those in uncertain situations (Bell and Cox, 2015). As an explanation for the present results, it may be possible that attentional salience is a necessary, but not sufficient condition, under which social norms exert their influence. Some other psychological motivation (e.g., uncertain situations) may also be necessary for the effects of social norms to emerge. Further research should explore such matters in the context of legal psychology and policing.

## Limitations

The inclusion of both professional police officers and police recruits was a sound decision because it was reasonable to assume many similarities between these populations (Gatto and Dambrun, 2012; Lander, 2013; Wieslander, 2019). However, it is possible that differential experiences between these groups could have influenced the findings. In addition, despite research (*ibid*.) which has indicated strong socialization processes, professional police and police recruits could be groups with differing normative codes of conduct. Another limitation concerns the measure of social identity which was implemented shortly after data collection had commenced. Consequently, 30 participants completed the study before implementation. Also, since it was a measure of identification with the police occupation, it could be biased toward professional police officers and possibly have excluded recruits. Another limitation was that the small sample size in Study 1 restrained any firm conclusions; however, as this was designed as a pilot study, we believe that Study 1 fulfilled its purpose. Further on, although Study 2 did not have statistical power to detect small effects, it had power to detect medium- to large-sized effects. Further research could aim at trying to gain more police participants and build upon this study.

## Implications and Future Directions

The present research did not examine police officers and recruit’s knowledge directly (i.e., *via* an explicit knowledge test). Future research should examine police knowledge explicitly by asking officers and recruits to complete a proper test of their knowledge regarding intoxicated witnesses’ ability to recall events. In addition, in the present studies, participants were not asked about what training in assessing alcohol-intoxication they may have received. This limited the scope of the discussion around potential issues with using observational methods to assess intoxication level. We encourage researchers to examine this in future studies.

The present findings cautiously suggest a potential vacancy in the Swedish police education. Not only are national guidelines for professional police required, but future research should also investigate this potential gap concerning alcohol-intoxication and witness memory in the curriculum at Swedish Police Academies. This should not be taken as an indication that police officers, departments, police recruits, or the academies are solely responsible for this potential deficiency. Researchers also carry a responsibility to share knowledge in an accessible manner where bridging this gap is paramount for scientific research to become relevant outside of the scientific community (see Hagsand et al., 2020; Hagsand, 2021).

The research-based message impacted the interview decision but not the perception of credibility and future research should investigate why this was the case. Still, participants embraced the content of the message and decided to interview in line with research recommendations. Therefore, future implementation of national policy guidelines regarding alcohol-intoxicated witnesses could be disseminated *via* an informative message. Since the effects of interventions have tended to be strongest directly after implementation (e.g., Fernandes et al., 2014), future research should investigate the long-term effects of providing police officers and recruits with research-based information.

Because some of our findings contradicted the general trend within the field, future research should attempt to replicate these findings and examine if there are any circumstances under which social norms could influence police decisions and perceptions. One possibility is that self-selection bias (i.e., which participants decided to take part in the studies) may have skewed some results. While this is a common issue in any research design, we still recommend that future research replicate these findings in other samples of the population.

Due to the novelty of the current study, many additional advances within the study design are made available for future research. For example, different genders could act as witness and interviewers, and instead of using video clips for assessment, participants could view face-to-face interactions between interviewer and witnesses, and additional dependent variables. We encourage other researchers to not only replicate the proposed study (e.g., making it more generalizable to other countries), but also add additional variables and make other adjustments to ultimately further the field of legal psychology in a meaningful direction.

## Conclusion

The present findings suggested that police officers and police recruits might make decisions in the absence of research-based knowledge, leading to inconsistent interview decisions, as well as their ability to deem witnesses as credible. The results also highlighted that a research-based message, in the shape of procedure guidelines, could be a way to align the decision to interview with research recommendations, but only when there is enough information included in the message, as just a single sentence might not work. Regardless of intoxication level, witnesses were perceived as less credible when under the influence, and this judgment yet again appeared to be made in the absence of scientific research. Furthermore, social norms were found to be ineffective to influence police on their decisions to interview, and this invites further investigation. The current findings added to the legal psychology literature (Evans et al., 2009; Crossland et al., 2018; Monds et al., 2021a; Hagsand et al., 2022) by showing that the perception of intoxicated witness as less credible than sober witness is present at BrAC levels as low as .04%. These findings also indicated that police officers and recruits may have a basic understanding that witnesses can be interviewed at low levels of alcohol-intoxication (i.e., around .04%), but that they believe that this ceases to be the case at intoxication levels lower than what scientific research has suggested. This novel examination on social norms and research-based messages in the context of police studies on alcohol-intoxicated witnesses could help to inform future research endeavors to continue to build upon this knowledge and examine this area more closely.

## Data Availability Statement

The datasets presented in this study can be found in online repositories. The names of the repository/repositories and accession number(s) can be found at Open Science Framework (OSF) https://osf.io/qv8tn/?view_only=eb195362d8ab49308e824dc4743bb3af.

## Ethics Statement

Ethical review and approval were not required for the study on human participants in accordance with the local legislation and institutional requirements. The patients/participants provided their written informed consent to participate in this study.

## Author Contributions

All authors contributed to the conceptualization of research questions, the study design, the data collecting procedure, and approved the submitted version. DP carried out quantitative analyses on both Study 1 and 2, wrote the original draft of the manuscript, and contributed to subsequent manuscript writing. MB helped in all stages of the research project, from formulating the research ideas and functioning as co-PI, to feedback on the data collection process, manuscript, and data-analysis. AH is the senior researcher who acquired funding for this project as PI, and she has overseen all stages of this research project, including the conceptualizing of research questions, data collection, and manuscript writing.

## Funding

This study was funded by grant from the Adlerbertska Research Foundation (Dnr GU 2020/751) at University of Gothenburg, Sweden. Part of this research (Study 2) has been presented at the virtual European Association of Psychology and Law (EAPL) conference in August 2021.

## Conflict of Interest

The authors declare that the research was conducted in the absence of any commercial or financial relationships that could be construed as a potential conflict of interest.

## Publisher’s Note

All claims expressed in this article are solely those of the authors and do not necessarily represent those of their affiliated organizations, or those of the publisher, the editors and the reviewers. Any product that may be evaluated in this article, or claim that may be made by its manufacturer, is not guaranteed or endorsed by the publisher.

## Footnotes

^1^Open-ended question format is an interview technique in which the interviewee is encouraged to provide a free-form answer. In contrast, a closed question often requires a yes or no answer or impose some other limit on possible answers. This was a design element used in the research from which the film material originated and was not pursued further in the present research.

^2^Data availability: All SPSS data sets and outputs can be downloaded from the Open Science Framework (OSF). https://osf.io/qv8tn/?view_only=eb195362d8ab49308e824dc4743bb3af

^3^View the complete research-based message at Open Science Framework (OSF): https://osf.io/qv8tn/?view_only=eb195362d8ab49308e824dc4743bb3af

^4^The original Qualtrics questionnaire (in Swedish) used in Study 2 can be viewed at the Open Science Framework (OSF): https://osf.io/qv8tn/?view_only=eb195362d8ab49308e824dc4743bb3af

^5^In Sweden, alcohol-intoxication is commonly referred to in permille (‰). However, except for the actual study data collection and this mention in the method section, the international convention of referencing intoxication as percent (%) was used throughout this manuscript.

^6^The present study violated some of the assumptions of the general linear model. While analysis of variance has demonstrated robustness against violations (e.g., Carifio and Perla, 2007; Norman, 2010), the use of parametric test with ordinal data has been a much-debated issue (Carifio and Perla, 2008). Unreported sensitivity analyses using the non-parametric Kruskal-Wallis test re-tested the main analyses and these results did not alter any of the parametric inferences.

^7^Data availability: All SPSS data sets and outputs can be downloaded from the Open Science Framework (OSF). https://osf.io/qv8tn/?view_only=eb195362d8ab49308e824dc4743bb3af
